# Serum calprotectin correlates stronger with inflammation and disease activity in ACPA positive than ACPA negative rheumatoid arthritis

**DOI:** 10.1093/rheumatology/kead641

**Published:** 2023-12-04

**Authors:** Kristina Sejersen, Tomas Weitoft, Ann Knight, Jörgen Lysholm, Anders Larsson, Johan Rönnelid

**Affiliations:** Department of Medical Sciences, Section of Clinical Chemistry, Uppsala University, Uppsala, Sweden; Unilabs AB, Stockholm, Sweden; Department of Research and Development, Uppsala University/Region Gävleborg, Gävle, Sweden; Section of Rheumatology, Department of Medical Sciences, Uppsala University Hospital, Uppsala, Sweden; Clinic of Rheumatology, Falun Hospital, Falun, Sweden; Department of Medical Sciences, Section of Clinical Chemistry, Uppsala University, Uppsala, Sweden; Department of Immunology, Genetics and Pathology, Uppsala University, Uppsala, Sweden

**Keywords:** rheumatoid arthritis, calprotectin, inflammation, anti-citrullinated peptide antibodies, antibodies against cyclic citrullinated peptide version 2, rheumatoid factor

## Abstract

**Objectives:**

The aim of the present study was to evaluate the performance of serum and SF levels of the granulocyte protein calprotectin as an inflammatory biomarker in RA patients with knee synovitis.

**Methods:**

Seventy-six RA patients with ongoing knee synovitis were included. Data on DAS with 28 joints and their subcomponents and radiological destruction of the affected knee were collected. White blood cell count, CRP, ACPA against cyclic citrullinated peptide version 2 (anti-CCP2), IgM RF and calprotectin were analysed in parallel in circulation and in SF. Counts of polynuclear and mononuclear cells were measured in SF.

**Results:**

Serum (S)-calprotectin correlated more strongly than SF-calprotectin with inflammatory markers and disease activity. Instead, SF-calprotectin showed a strong correlation to SF counts of white blood cells, and especially to polymorphonuclear cell counts (Spearman’s ρ = 0.72, *P* < 0.001). S-calprotectin showed markedly stronger correlation with inflammatory markers and disease activity in ACPA positive as compared with ACPA negative RA patients; a similar difference was observed for patients with and without IgM RF.

**Conclusion:**

The particularly strong association between circulating calprotectin and inflammation in ACPA positive RA is a new argument for a specific role for polymorphonuclear granulocytes/neutrophils in this RA subset. Measurement of calprotectin in SF does not convey any additional benefit compared with measurement in the circulation in RA patients with knee synovitis.

Rheumatology key message

- Anti-CCP2 positive RA has a strong association between calprotectin and extent of clinical disease.

## Introduction

RA is a chronic, inflammatory joint disease that has complex immune mediated pathogenesis [1]. In order to optimize and individualize the treatment of patients with RA, it is important to improve knowledge on early biomarkers predicting more aggressive course of the disease with higher levels of inflammation. The calprotectin level in circulation has many characteristics of such a biomarker. Calprotectin is a major constituent of neutrophils/polymorphonuclear granulocytes, cell types previously shown to participate in the pathogenesis of autoimmune inflammatory diseases including RA [2]. Calprotectin is a protein present in the cytosol of neutrophils, constituting 40–60% of cytosolic protein, as well as being a major monocyte/macrophage protein belonging to the S100 multigene subfamily of cytoplasmic EF-hand Ca^2+^-binding proteins. It is rapidly released upon neutrophil activation and is also a mediator of inflammation. For this reason, circulating calprotectin has gained interest as an inflammatory marker in RA [3–6]. Recent data show calprotectin to respond rapidly, within 2 h, to induction of inflammation [7]. Studies have found associations between calprotectin and disease activity in different rheumatic joint diseases [8–13], and it has been demonstrated that calprotectin levels correlated strongly with radiographic joint damage in patients with RA [14]. Calprotectin levels are elevated in both serum/plasma and SF in RA [15, 16], and calprotectin has been demonstrated in macrophages and fibroblast-like synoviocytes of the synovium [17, 18]. However, no significant correlation was observed between calprotectin in plasma and calprotectin in SF in patients with reactive arthritis. Further, there was no significant correlation between calprotectin concentration and white blood cell (WBC) counts in blood and SF [11].

Despite numerous previous demonstrations on associations between calprotectin and disease activity and severity in RA, we still lack a deeper understanding how of calprotectin relates to inflammation in patients with and without the autoantibodies used in RA classification: IgM RF and ACPA, which could potentially be useful for designing individualized therapies. For this reason, we set out to analyse calprotectin levels in serum as well as in SF in patients with RA with concomitant knee synovitis.

## Methods

### Study population

A total of 76 patients (17 [22%] men and 59 [78%] women) with RA and ongoing knee synovitis in the outpatient rheumatology departments at the hospitals at Gävle, Falun and Uppsala were included in this observational study [19, 20]. RA according to the 1987 ACR classification criteria [21] and signs and symptoms of knee synovitis constituted inclusion criteria for the study. Exclusion criteria were patients in function class 4 according to Steinbrocker *et al*. [22] and patients receiving oral corticosteroid (CS) treatment corresponding to >10 mg/day of prednisolone. The number of tender and swollen joints was counted (TJC and SJC, respectively), general health (GH) and disease activity were measured by a visual analogue scale (VAS), and DAS with 28 joints (DAS28) [23] was calculated using either ESR or CRP as a laboratory inflammatory marker. SF was aspirated from the knee in parallel with serum (S) sampling. A radiographic examination of the aspirated knee was performed, and joint destruction was graded between 0 and 5 according to Larsen and Dale [24] by an independent radiologist. The Swedish version of the Health Assessment Questionnaire (HAQ) [25] was used to evaluate the level of disability.

### Laboratory methods

Serum and SF samples were centrifuged within 1 h for 20 min at 1800 *g* and stored at −70°C until analysis [19].

White blood cell (WBC) counts in blood (B-WBC) and SF, and count of polynuclear neutrophils (PMN) and mononuclear cells (MNC) in SF were analysed using a Sysmex XN instrument (Sysmex, Kobe, Japan). ESR in blood (B-ESR) was analysed using the manual Westergren method or automated Westergren method using Starrsed Interrliner RL (Mechatronics, Zwaag, The Netherlands). CRP was analysed on an Architect ci8200 (Abbot Laboratories, Abbott Park, IL, USA).

Fluorescence enzyme immunoassays were performed on a Phadia250 instrument (Thermo Fisher Scientific, Uppsala, Sweden) to analyse antibodies against cyclic citrullinated peptide version 2 (anti-CCP2) and IgM RF with cutoffs of 7 AU/ml and 5 IU/ml, respectively. Analysis of calprotectin was performed with a particle enhanced turbidimetric assay (Gentian AS, Moss, Norway) on a Mindray BS200 instrument (Mindray, Shenzhen, China).

### Statistical analyses

All non-parametric statistical analyses were performed using Analyse-it for Excel (Analyse-it Software, Leeds, UK) and data displayed as median, mean, minimum and maximum, with categorical variables were expressed as number (%). Correlations were calculated using Spearman’s rank order correlation tests using SPSS Statistics version 26 (IBM Corp., Armonk, NY, USA). A two-sided *P* < 0.05 was considered significant. The correlation was considered as weak for 0.1 < ρ < 0.39, moderate for 0.4 ≤ ρ < 0.69, and strong for ρ ≥ 0.7 [26].

### Ethics

The study was approved by the National Ethical Review Agency (EPM) (No. EPN U2007-03-07, Dnr 2007/047). The Declaration of Helsinki and its subsequent revisions were followed. Patients were included in the study after written informed consent was obtained from the patients.

## Results

### Baseline characteristics

Patient mean and median age were 69 and 62 years, respectively (24–87), median weight 72 (44–119) kg, and their median RA duration 9 (0–60) years. Twenty-five of the patients (33%) were current and 13 (17%) previous smokers. Sixty-six (86%) of the patients were treated with methotrexate alone or in combination with other DMARDs. Total baseline characteristics for the full group and the autoantibody-defined subgroups are shown in Table 1.

**Table 1.** Basal and clinical characteristics and medications of patients with RA

Characteristic/medication	Patient group				
	All patients (*n* = 76)	Anti-CCP2 positive patients (*n* = 55)	Anti-CCP2 negative patients (*n* = 21)	IgM RF positive patients (*n* = 53)	IgM RF negative patients (*n* = 23)
Age, median (IQR), years	62 (51–71)	61 (52–70)	63 (43–71)	62 (54–70)	61 (41–72)
Sex, *n* (%)					
Male	17 (22)	12 (22)	5 (24)	10 (19)	7 (30)
Female	59 (78)	43 (78)	16 (76)	43 (81)	16 (70)
Weight, median (IQR), kg	72 (61–83)	71 (61–84)	73 (66–82)	71 (61–83)	73 (66–81)
Smoker, *n* (%)					
Never	38 (50)	30 (55)	8 (38)	30 (57)	8 (35)
Ever	25 (33)	18 (33)	7 (33)	16 (30)	9 (39)
Current	13 (17)	7 (13)	6 (29)	7 (13)	6 (26)
RA duration, median (IQR), years	9 (2–19)	10 (3–20)	8 (2–16)	10 (3–20)	6 (2–13)
Site off knee arthritis, *n* (%)					
Single knee	70 (92)	50 (91)	20 (95)	47 (89)	23 (100)
Both knees^a^	6 (8)	5 (9)	1 (5)	6 (11)	0 0
DMARD, *n* (%)					
Monotherapy cDMARD^b^	48 (63)	36 (65)	12 (57)	34 (64)	14 (61)
Monotherapy bDMARD^c^	5 (7)	3 (5)	2 (10)	4 (8)	1 (4)
Combination of cDMARDs	6 (8)	5 (9)	1 (5)	5 (9)	1 (4)
Combination of cDMARD and bDMARD	7 (9)	4 (7)	3 (14)	3 (6)	4 (17)
No DMARD	10 (13)	7 (13)	3 (14)	7 (13)	3 (13)
Prednisolone daily dose, *n* (%)					
2.5 mg	4 (5)	4 (7)	0 0	4 (8)	0 0
5 mg	18 (24)	11 (20)	7 (33)	11 (21)	7 (30)
7.5 mg	5 (7)	3 (5)	2 (10)	2 (4)	3 (13)
10 mg	3 (4)	2 (4)	1 (5)	2 (4)	1 (4)
None	46 (61)	35 (64)	11 (52)	34 (64)	12 (52)

a Aspirated with at least 1 month interval.

b Methotrexate, leflunomide, azathioprine, chloroquine, sodium aurothiomalate, sulfasalazine.

c Etanercept, adalimumab, tocilizumab. bDMARD: biologic DMARD; cDMARD: conventional DMARD; IQR: interquartile range.

### S-calprotectin in the whole RA study population and in autoantibody-defined subgroups

In an initial whole study population analysis, S-calprotectin was found to have strong correlation with S-CRP (ρ = 0.78, *P* < 0.001) and moderate correlation with B-ESR (ρ = 0.50, *P* < 0.001), DAS28-CRP (ρ = 0.61, *P* < 0.001), SF-calprotectin (ρ = 0.60, *P* < 0.001), SF-CRP (ρ = 0.60, *P* < 0.001), B-WBC (ρ = 0.58, *P* < 0.001), SF-PMN (ρ = 0.57, *P* < 0.001), DAS28 (ρ = 0.53, *P* < 0.001), SJC (ρ = 0.47, *P* < 0.001) and SF-WBC (ρ = 0.47, *P* < 0.001). Weak correlation for S-calprotectin was observed with anti-CCP2 (ρ = 0.33, *P* < 0.05), HAQ (ρ = 0.30, *P* < 0.05), S-IgM-RF (ρ = 0.27, *P* < 0.05) and TJC (ρ = 0.24, *P* < 0.05). No significant correlation was found between S-calprotectin and SF-MNC, S-IgA, S-IgG, S-IgM, SF-IgG, GH global VAS and Larsen–Dale index (Table 2).

**Table 2.** Correlation between S-calprotectin and other biomarkers of inflammation/disease severity

Variable	Patient group													
	All patients						Anti-CCP2 positive patients				Anti-CCP2 negative patients			
	*n*	Median	Min	Max	**Spearman’s ρ**	*P*-value (2-tailed)	*n*	Median	Spearman’s ρ	*P*-value (2-tailed)	*n*	Median	Spearman’s ρ	*P*-value (2-tailed)
S-calprotectin, mg/l	76	1.84	0.26	28.02			55	2.03			21	1.61		
SF-calprotectin, mg/l	72	20.4	0.1	136.8	0.60	<0.00001**	51	26.6	0.62	<0.00001**	21	19.8	0.50	0.02099*
B-WBC, 10^9^/l	36	8.2	3.5	15.3	0.58	0.00019**	26	8.2	0.55	0.00360*	10	7.9	0.61	0.05997
SF-WBC, 10^9^/l	63	3.2	0	152.1	0.47	0.00012**	44	2.8	0.55	0.00011**	19	3.2	0.27	0.25645
SF-PMN, 10^9^/l	57	1.6	0	32.1	0.57	<0.00001**	39	1.6	0.61	0.00004**	18	1.3	0.51	0.03028*
SF-MNC, 10^9^/l	57	1.6	0	132.6	0.24	0.07125	39	1.4	0.37	0.01923*	18	2	0.06	0.82607
B-ESR, mm	72	26	2	97	0.50	0.00001**	52	36	0.54	0.00004**	20	18	0.31	0.18054
S-CRP, mg/l	76	13	1	146	0.78	<0.00001**	55	13	0.82	<0.00001**	21	12	0.58	0.00580*
SF-CRP, mg/l	76	13	0	94	0.60	<0.00001**	55	13	0.62	<0.00001**	21	14	0.58	0.00550*
S-IgA, g/l	69	2.7	1.2	5.7	0.07	0.55969	51	2.7	0.09	0.52876	18	2.3	−0.04	0.86121
S-IgG, g/l	75	11.3	5.7	30.1	0.03	0.82681	55	11.0	−0.00	0.99750	20	14.4	0.20	0.40882
SF-IgG, g/l	76	4.0	0.8	21.8	0.05	0.67794	55	4.6	0.06	0.67168	21	3.7	−0.08	0.73708
S-IgM, g/l	69	1.0	0	6.7	0.04	0.74676	51	0.9	0.13	0.37522	18	1.2	−0.20	0.43306
Anti-CCP2, U/ml	76	126	1.1	5041	0.33	0.00407*	55	330	0.42	0.00161*	21	1.7	−0.10	0.66287
S-IgM-RF, U/ml	76	34.5	0	797.3	0.27	0.01714*	55	71	0.40	0.00232*	21	0.9	0.04	0.86218
DAS28	76	4.34	2.00	7.64	0.53	<0.00001**	55	4.48	0.54	0.00002**	21	4.20	0.37	0.10120
DAS28-CRP	71	4.09	2.05	7.02	0.61	<0.00001**	52	3.81	0.65	<0.00001**	19	4.14	0.41	0.08227
GH global VAS	71	53	0	100	0.21	0.07773	52	53	0.21	0.14431	19	50	0.25	0.31038
SJC	72	2	1	15	0.47	0.00003**	52	2	0.60	<0.00001**	20	3	0.21	0.37891
TJC	72	2	0	15	0.24	0.0423*	52	2	0.28	0.04324*	20	3	0.40	0.08371
Larsen–Dale index, 0–5	70	1	0	5	0.02	0.87625	52	1	−0.02	0.86582	18	1	0.19	0.45587
HAQ	76	1.00	0	2.63	0.30	0.00795*	55	1.00	0.33	0.01473*	21	0.88	0.30	0.18691

Correlations were made for whole RA population, and for RA anti-CCP2 positive and RA anti-CCP2 negative patients. Degrees of correlation (ρ coefficient) are given as Spearman’s ρ value with indication of degree of significance:

* *P* < 0.05,

** *P* < 0.001; no asterisk signifies no statistical significance. DAS28: DAS for 28 joints; GH: general health; MNC: mononuclear cells; PMN: polynuclear neutrophils; SJC: swollen joint count; TJC: total joint count; VAS: visual analogue scale; WBC: white blood cell.

In order better to understand if S-calprotectin is correlated with clinically used markers to define subgroups of RA, we performed sub-analyses of RA patients positive or negative for anti-CCP2 (Table 2) and positive or negative for RF (Table 3).

**Table 3.** Correlation between S-calprotectin and other biomarkers/disease severity in S-IgM RF positive and negative patients

Variable	Patient group							
	IgM RF positive patients				IgM RF negative patients			
	*n*	Median	Spearman’s ρ	*P*-value (2-tailed)	*n*	Median	Spearman’s ρ	*P*-value (2-tailed)
S-calprotectin, mg/l	53	2.67			23	1.61		
SF-Calprotectin, mg/l	49	28.3	0.64	<0.00001**	23	15.3	0.40	0.05806
B-WBC, 10^9^/l	22	8.0	0.67	0.00071**	14	8.7	0.44	0.11535
SF-WBC, 10^9^/l	42	3.3	0.52	0.00039**	21	3.2	0.31	0.17029
SF-PMN, 10^9^/l	37	1.7	0.61	0.00007**	20	0.9	0.42	0.06775
SF-MNC, 10^9^/l	37	1.6	0.31	0.06415	20	1.9	0.19	0.43351
B-ESR, mm	50	32	0.51	0.00015**	22	18	0.37	0.08582
S-CRP, mg/l	53	13	0.84	<0.00001**	23	12	0.59	0.00312*
SF-CRP, mg/l	53	13	0.59	<0.00001**	23	14	0.66	0.00059**
S-IgA, g/l	49	2.7	0.08	0.58028	20	2.6	0.00	0.98494
S-IgG, g/l	53	11.3	0.02	0.88743	22	11.0	0.12	0.58340
SF-IgG, g/l	53	4.0	0.09	0.50990	23	4.3	−0.14	0.51710
S-IgM, g/l	49	0.9	0.10	0.49699	20	1.2	−0.13	0.59565
Anti-CCP2, U/ml	53	310	0.45	0.00069*	23	2	−0.03	0.90260
S-IgM-RF, U/ml	53	92	0.37	0.00642*	23	0.9	−0.20	0.37172
DAS28	53	4.50	0.48	0.00029**	23	4.05	0.56	0.00517*
DAS28-CRP	50	3.91	0.62	<0.00001**	21	4.14	0.59	0.00483*
GH global VAS	50	53	0.11	0.45599	21	51	0.49	0.02567*
SJC	50	3	0.54	0.00005**	22	2	0.36	0.10117
TJC	50	2	0.24	0.09857	22	3	0.40	0.06815
Larsen–Dale index, 0–5	50	1	0.02	0.90805	20	1	0.08	0.74154
HAQ	53	1.00	0.23	0.09488	23	0.88	0.49	0.01790*

Degrees of correlation (ρ coefficient) are given as Spearman’s ρ value with indication of degree of significance:

* *P* < 0.05,

** *P* < 0.001; no asterisk signifies no statistical significance. DAS28: DAS for 28 joints; GH: general health; MNC: mononuclear cells; PMN: polynuclear neutrophils; SJC: swollen joint count; TJC: total joint count; VAS: visual analogue scale; WBC: white blood cell.

To be noted, subdividing the study population into patients positive or negative for anti-CCP2 revealed significant very strong correlation between S-calprotectin and S-CRP in RA patients positive for anti-CCP2 (Table 2). In RA patients negative for anti-CCP2 (anti-CCP2 negative RA), more moderate correlations were observed between S-calprotectin and with S-CRP. There was also moderate correlation between S-calprotectin and SF-CRP, SF-calprotectin and SF-PMN in both anti-CCP2 positive and negative RA.

Moderate correlations were found between S-calprotectin and B-WBC, SF-WBC, B-ESR, S-anti-CCP2, S-IgM-RF, DAS28-CRP, DAS28 and SJC, and weak correlation observed between S-calprotectin and SF-MNC, HAQ and TJC in patients with anti-CCP2 positive RA (Table 2).

Sub-analysis of RA patients according to presence or absence of RF revealed a strong correlation of S-calprotectin with S-CRP in patients with S-RF positive (Table 3). In RF negative patients a moderate correlation was observed between S-calprotectin and S-CRP (Table 3). A moderate correlation was also found between S-calprotectin and SF-CRP, DAS28, and DAS28-CRP in both S-IgM RF positive and negative RA (Table 3). Moderate correlations were found between S-calprotectin and SF-calprotectin, B-WBC, SF-PMN, SF-WBC, B-ESR, S-antiCCP2 and SJC, and weak correlation observed between S-calprotectin and S-IgM RF in patients with RF positive RA. There is a moderate correlation between S-calprotectin and HAQ and GH global VAS in patient with RF negative RA (Table 3).

### SF-calprotectin in whole RA study population and RA subgroups (RF, anti-CCP2)

To investigate if levels of calprotectin in synovial fluid would correlate better to other more widely used inflammatory markers for RA disease activity than S-calprotectin, we separately analysed SF-calprotectin. However, we found S-calprotectin to correlate more strongly than SF-calprotectin with markers of disease activity (Table 4).

**Table 4.** Correlation between SF-calprotectin and other inflammatory biomarkers/disease severity

Variable	Patient group													
	All patients						Anti-CCP2 positive patients				Anti-CCP2 negative patients			
	*n*	Median	Min	Max	Spearman’s ρ	*P*-value (2-tailed)	*n*	Median	Spearman’s ρ	*P*-value (2-tailed)	*n*	Median	Spearman’s ρ	*P*-value (2-tailed)
B-WBC, 10^9^/l	33	8.2	3.5	15.3	0.52	0.00171*	23	8.2	0.46	0.02599*	10	7.9	0.61	0.05997
SF-WBC, 10^9^/l	62	3.2	0	152.1	0.64	<0.00001**	43	3.1	0.75	<0.00001**	19	3.2	0.39	0.09733
SF-PMN, 10^9^/l	56	1.7	0	32.1	0.72	<0.00001**	38	1.7	0.82	<0.00001**	18	1.3	0.44	0.06720
SF-MNC, 10^9^/l	56	1.7	0	132.6	0.27	0.04043*	38	1.5	0.37	0.02333*	18	2.0	0.20	0.43282
B-ESR, mm	69	28	2	97	0.23	0.05907	49	40	0.29	0.04603*	20	18	0.04	0.85482
S-CRP, mg/l	72	14	1	146	0.47	0.00004**	51	14	0.50	0.00019**	21	12	0.31	0.17423
SF-CRP, mg/l	72	14	1	94	0.51	<0.00001**	51	14	0.55	0.00003**	21	14	0.47	0.03119*
S-IgA, g/l	65	2.6	1.2	5.7	0.16	0.21003	47	2.7	0.24	0.10093	18	2.3	−0.04	0.88043
S-IgG, g/l	71	11.3	4.1	30.1	0.06	0.63775	51	11.0	0.01	0.94949	20	14.4	0.20	0.40515
SF-IgG, g/l	72	4.4	0.8	21.8	0.34	0.00306*	51	5.1	0.31	0.02729*	21	3.7	0.40	0.07138
S-IgM, g/l	65	1.0	0	6.7	0.22	0.07780	47	0.9	0.29	0.04764*	18	1.2	0.11	0.67464
Anti-CCP2, U/ml	72	143	1.1	5041	0.12	0.30757	51	340	0.21	0.13649	21	1.7	−0.45	0.04037*
S-IgM-RF, U/ml	72	38.5	0	797.3	0.15	0.21762	51	92	0.25	0.07973	21	0.9	−0.19	0.41767
DAS28	72	4.45	2.00	7.64	0.31	0.00915*	51	4.55	0.27	0.05202	21	4.20	0.29	0.20500
DAS28-CRP	68	4.12	2.05	7.02	0.37	0.00207*	49	3.95	0.38	0.00771*	19	4.14	0.53	0.02062*
GH global VAS	68	54	0	100	0.06	0.63265	49	54	−0.08	0.57880	19	50	0.42	0.07188
SJC	69	3	1	15	0.32	0.00727*	49	3	0.40	0.00460*	20	3	0.22	0.36123
TJC	69	2	0	15	0.15	0.22573	49	2	0.10	0.50556	20	3	0.30	0.19568
Larsen–Dale index, 0–5	67	1	0	5	0.12	0.33373	49	1	0.07	0.61275	18	1	0.28	0.26473
HAQ	72	1.00	0	2.63	0.08	0.52076	51	1.00	0.05	0.72054	21	0.88	0.08	0.72354

Correlation between SF-calprotectin and other inflammatory biomarkers/disease severity in the whole RA population, and in patients with and without anti-CCP2. Degrees of correlation (ρ coefficient) are given as Spearman’s ρ value with indication of degree of significance:

* *P* < 0.05,

** *P* < 0.001; no asterisk signifies no statistical significance. DAS28: DAS for 28 joints; GH: general health; MNC: mononuclear cells; PMN: polynuclear neutrophils; SJC: swollen joint count; TJC: total joint count; VAS: visual analogue scale; WBC: white blood cell.

SF-calprotectin correlated strongly with SF-PMN and SF-WBC, and showed moderate correlation with SF-CRP, S-CRP, B-WBC and SJC. A weak correlation was also detected between SF-calprotectin and SF-MNC, SF-IgG, B-ESR and S-IgM in patient with anti-CCP2 positive RA.

In patients with anti-CCP2 negative RA, SF-calprotectin correlated moderately with SF-CRP and DAS28-CRP (Table 4). No association was detected between S-/SF-calprotectin levels and RA duration or therapeutic effect duration defined as days from intra-articular glucocorticoid injection to relapse (data not shown). Also, no correlation was observed between degree of radiological damage and SF-calprotectin levels.

## Discussion

In this study of patients with RA and knee synovitis, major findings were that S-calprotectin correlated better with markers of disease activity than SF-calprotectin, and a novel finding that S-calprotectin showed a highly significant and strong correlation with inflammatory markers in the anti-CCP2 positive subgroup of patients, stronger than in the anti-CCP2 negative subgroup.

The finding that anti-CCP2 positive RA has a much stronger association between calprotectin and extent of clinical disease than anti-CCP2 negative RA has, to our knowledge, never been observed before. The finding fits well with the concept of RA as two different sub-entities of the disease with different pathogeneses. Pathogenetically, anti-CCP positive RA is its own disease, separate from the antibody-negative group [27, 28]. Polymorphonuclear cells are suggested to have a central role in the emergence of anti-CCP by citrullination of histones in neutrophil extracellular traps (NETs) that could induce antibodies against citrulline residues [29–32]. As calprotectin is a major constituent of PMN, released early upon PMN activation, we speculate that calprotectin has a special role in anti-CCP2 positive RA. Calprotectin is classified as a damage-associated molecular pattern (DAMP) molecule. Neutrophils and monocytes are found at inflammatory sites, being among the first cells recruited, and are crucial for pathogen killing by an array of mechanisms, including production of cytokines, chemokines, reactive oxygen species, nicotinamide adenine dinucleotide phosphate-oxidase (NADPH oxidase), release of cytotoxic products and formation of NETs. Neutrophil activation was initially observed as induced by pathogens, and has subsequently been demonstrated to be induced also by endogenous ligands, DAMPs or alarmins [33], activated via the Toll-like receptor 4 pathway. This results in the production of TNF-α, IL-6 and other inflammatory cytokines [34]. High concentrations of calprotectin extracellularly are found at local sites of inflammation and in the serum of patients with autoimmune or inflammatory diseases (e.g. RA, inflammatory bowel disease or cystic fibrosis) [35–37], and represent a sensitive inflammatory biomarker strongly related to disease activity [38]. We have in this study chosen to use a turbidimetric immunoassay for S-calprotectin, which is a random access type enabling quick analysis of results, and further that the method can be applied to the various already existing instrument platforms present in hospital laboratories [39].

Further, we find it noteworthy that S-calprotectin had a moderate correlation with SJC both in anti-CCP2 positive and RF positive patients, but not in anti-CCP2 negative or RF negative patients. One plausible reason for this, namely that patients positive for RF or anti-CCP2 would have higher SJC, appears not to be a satisfactory explanation of our finding, since SJC is comparable in antibody positive and antibody negative subgroups.

Our results also demonstrates that S-calprotectin correlates more strongly than SF-calprotectin with markers of inflammation and signs of clinical disease activity in RA. The finding that the association is stronger for S-calprotectin than for SF-calprotectin is not entirely new [11], and is probably at least partly due to granulocytes disintegration in the test tube before SF separation. Our interpretation of these results is that analysis of SF-calprotectin will not add clinical value to analysis of calprotectin in a clinical setting.

To conclude, the particularly strong association between circulating calprotectin and inflammation in ACPA positive RA is a new argument for a specific role for polymorphonuclear granulocytes/neutrophils in this RA subset. Measurement of calprotectin in SF does not convey any additional benefit compared with measurement in the circulation in RA patients with knee synovitis.

### Limitations and strengths

There are several limitations of the present study, including a cross-sectional design with only North European patients, small sample size, unequal gender distribution and lack of coverage of younger age groups. The small sample size also precluded multivariate analyses, e.g. with adjustments for age, sex and treatment. Strengths of our study are that we have a cohort with parallel blood and synovial fluid samples paired with accurate clinical and laboratory characteristics from the time of examination, and measurement of SF-calprotectin.

## Data Availability

All relevant data underlying this article are available in the article.
